# Prognostic value of fibrinogen-to-albumin ratio combined with coronary calcification score in patients with suspected coronary artery disease

**DOI:** 10.1186/s12872-023-03193-z

**Published:** 2023-04-04

**Authors:** Xin-Xin Tian, Jun-Yi Luo, Fen Liu, Ya-Jing Qiu, Fan Luo, Lu Zeng, Zhuo-Ran Zhang, Yi-Ning Yang, Xiao-Mei Li

**Affiliations:** 1 Department of Cardiology, the First Affiliated Hospital of Xinjiang Medical University, 137 Liyushan South Road, Urumqi, 830054 Xinjiang China; 2 State Key Laboratory of Pathogenesis, Prevention and Treatment of High Incidence Diseases in Central Asia, Clinical Medical Research Institute, The First Affiliated Hospital of Xinjiang Medical University, Urumqi, China; 3 Department of Cardiology, People’s Hospital of Xinjiang Uygur Autonomous Region, Urumqi, China

**Keywords:** Coronary calcification score, Fibrinogen-to-albumin ratio, Coronary artery disease, Cardiovascular events

## Abstract

**Objective:**

The aim of this work was to evaluate the predictive value of FAR combined with CACS for MACCEs.

**Background:**

The fibrinogen-albumin-ratio (FAR), a novel biomarker of inflammation, is associated with the severity of coronary artery disease (CAD). Coronary calcification score (CACS) is associated with the severity of coronary stenosis and is closely related to the prognosis of CAD patients. What is the prognostic value of FAR in patients with chest pain, which has not been reported. This study aims to evaluate the relationship between CACS and FAR and their impact on prognosis in patients with suspected CAD.

**Methods:**

We used information from 12,904 individuals who had coronary computed tomography angiography (CTA) for chest pain and tracked down any significant adverse cardiac and cerebrovascular events (MACCEs). The following formula was used to calculate FAR: fibrinogen (g/L)/albumin (g/L). Patients were separated into groups with greater levels of FAR (FAR-H) and lower levels of FAR (FAR-L) in accordance with the ideal cut-off value of FAR for MACCEs prediction. In addition, patients were divided into three groups based on their CACS scores (CACS ≤ 100, 100 < CACS ≤ 400, and CACS > 400).

**Results:**

4946 patients [62(55–71) years, 64.4% male] were ultimately enrolled in the present study. During follow-up, a total of 234 cases (4.7%) of MACCEs were documented. Linear regression analysis results showed that CACS (R2 = 0.004, Standard β = 0.066, *P* < 0.001) was positively associated with FAR in patients with chest pain.Compared to ones with FAR-L, FAR-H had an increased risk for MACCEs (adjusted HR 1.371(1.053–1.786) *P* = 0.019). Multivariate Cox regression showed that age (adjusted HR 1.015 95% CI 1.001–1.028;*p* = 0.03), FAR (adjusted HR 1.355 95% CI 1.042–1.763;*p* = 0.023),FBG (adjusted HR 1.043 95% CI 1.006–1.083;*p* = 0.024) and CACS (adjusted HR 1.470 95% CI 1.250–1.727;*p* < 0.001) were the independent risk factors for MACCEs. The FAR and CACS significantly improved MACCEs risk stratification, contributing to substantial net reclassification improvement ( NRI 0.122, 95% CI 0.054–0.198, *P* < 0.001) and integrated discrimination improvement(IDI 0.011, 95% CI 0.006–0.017, *P* < 0.001).

**Conclusion:**

FAR was an independent risk factor for MACCEs. The results showed that CACS was positively associated with FAR in patients with suspected CAD. A higher level of FAR and heavier coronary calcification burden was associated with worse outcomes among patients with suspected CAD. FAR and CACS improved the risk identification of patients with suspected CAD, leading to a significant reclassification of MACCEs.

**Supplementary Information:**

The online version contains supplementary material available at 10.1186/s12872-023-03193-z.

## Introduction

Chest pain is a common outpatient and emergency symptom in clinic. Determining whether chest pain is caused by coronary artery disease (CAD) is essential to guide appropriate symptomatic and preventive treatment.Coronary computed tomography angiography (CTA) plays an important role in the diagnosis of patients with suspected CAD [1, 2]. Coronary CTA provides prognostic information for the prediction of future cardiovascular events and the incidence of major adverse cardiovascular events (MACE) increases with the Coronary calcification (CAC) burden [3, 4]. Coronary calcification score (CACS) is associated with the severity of coronary stenosis and is closely related to the prognosis of patients with CAD [5, 6]. Previous studies have shown that various inflammatory biomarkers are associated with the severity and prognosis of CAD [7].

The relationship between serum albumin content and inflammatory and hemostatic processes has been documented [8]. Additionally, albumin could inhibit platelet activation [9]. A risk factor for CAD, particularly myocardial infarction (MI), is hypoproteinemia [10]. Fibrinogen, which is created by the liver, is biomarker of the inflammatory response and a procoagulant status indicator [11]. According to studies, plasma fibrinogen levels can indicate cardiovascular events in the general population and are related to how severe CAD is [12]. Fibrinogen and albumin are therefore regarded as key indicators of inflammatory transformation. Recent research found that the presence of FAR was closely linked to the severity of CAD [13, 14] and to being a known prognostic factor for breast cancer and liver cancer [15, 16]. In addition, In a cohort study, higher FAR levels were associated with worse 5-year outcomes in patients with coronary heart disease undergoing percutaneous coronary intervention [13].

CACS and FAR reflect plaque load and inflammatory status, respectively. We speculate that FAR and CACS can better identify high-risk individuals in patients with suspected coronary heart disease, and more accurate risk assessment should be performed in these populations.However, no studies have investigated the relationship between CACS and FAR in patients with suspected CAD. In addition, the prognostic value of FAR is not clear for patients with suspected CAD. In the light of the above, the present study was conducted to evaluate the relationship between FAR and CACS, and further determine the joint effect of FAR and CACS in patients with suspected coronary heart disease.

## Methods

### Study patients

**Fig. 1:**
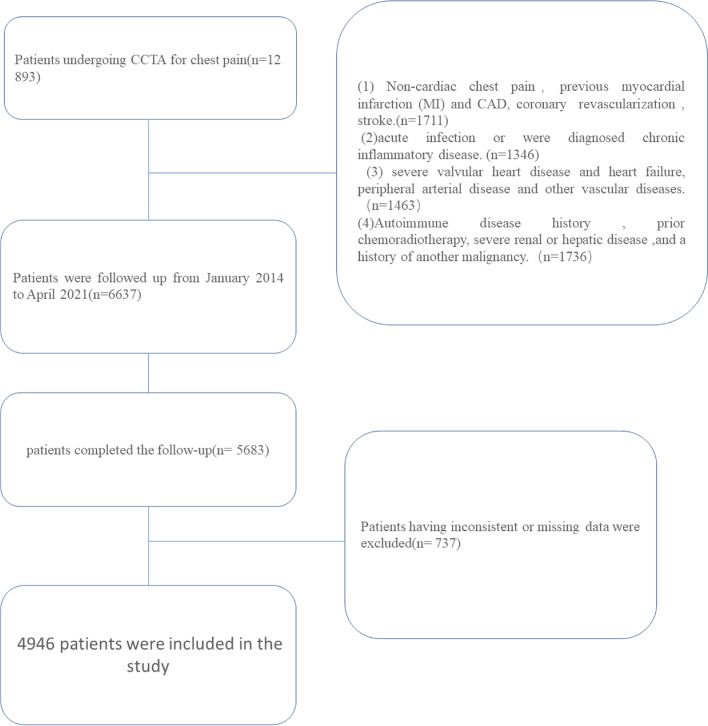
Flow chart for the inclusion and exclusion of study patients

We used the data of 12,893 patients undergoing Coronary CTA for chest pain to perform the study. Patients were enrolled following described exclusion criteria: (1) Non-cardiac chest pain, previous myocardial infarction (MI) and CAD, coronary revascularization, or stroke. (*n* = 1711). (2) acute infection or were diagnosed with chronic inflammatory disease (*n* = 1346). (3) severe valvular heart disease and heart failure, peripheral arterial disease, and other vascular diseases. (*n* = 1463). (4) Autoimmune disease history, prior chemoradiotherapy, severe renal or hepatic disease,and a history of another malignancy (*n* = 1736) (Fig. 1). After excluding, the 6637 patients with chest pain who were followed up from January 2014 to April 2021 by telephone or outpatient clinical visit each year, 5683 (85.7%) patients completed the follow-up. Patients having inconsistent or missing data were excluded (*n* = 737). Finally, a total of 4946 patients were included in the study. The study was approved by the ethics committee of the First Affiliated Hospital of Xinjiang Medical University.

### Endpoints and follow-up

The primary endpoint was a major adverse cardiac and cerebrovascular events (MACCEs). MACCEs were defined as cardiac death, Non-fatal MI, stroke, all-cause death, or revascularization. After the coronary CTA, follow-up for adverse clinical outcomes until the occurrence of an endpoint of interest or end of follow-up (April 2021), whichever came first.

### Coronary CTA acquisition and analysis

Those CTA were uniformly acquired by using multi-detector row CT scanners consisting of 64-rows or greater. CACS were measured by using the scoring system developed by Agatston [17]. For this investigation, we evaluated the severity of coronary artery calcification from three different groups (CACS ≤ 100, 100 < CACS ≤ 400, and CACS > 400).

### Data collection and risk factors definitions

Clinical information was gathered from all of the medical records by trained clinicians who were unaware of the study's objectives. The information included information on age, gender, smoking history, family history of CAD, history of diabetes and hypertension, and blood pressure (SBP and DBP) and CACS. Upon admission, peripheral venous blood samples were taken following an overnight fast, and they were examined promptly after sampling. The following measurements were examined: albumin, fibrinogen, uric acid, creatinine, triglyceride, high-density lipoprotein cholesterol (HDL-C), low-density lipoprotein cholesterol (LDL-C), fasting blood glucose (FBG), and total cholesterol (TC).The FAR was calculated from serum fibrinogen and albumin concentrations. All of the patients underwent the first Coronary CTA during this hospitalization. The estimated glomerular filtration rate (eGFR) was calculated by the Modification of Diet in Renal Disease (MDRD) formula [18].

### Statistics

Categorical variables were analyzed as frequencies with percentages. Continuous variables that do not conform to the normal distribution are expressed as the median value (25th to 75th percentile), and other continuous variables that conform to the normal distribution are expressed as the mean and standard deviation (SD) values. Mann Whitney U test was used to assess differences in continuous variables that did not meet the assumption of a normal distribution. Comparison of categorical variables among different groups was analyzed by Chi-square test or Fisher’s exact test, as appropriate. Univariate and multivariate stepwise Cox proportional hazard regression analyses were constructed to identify the independent predictors of MACCEs. Pearson correlation and linear regression analysis were constructed to evaluate the cor-relation between FAR and CACS. We selected the optimum cutoff for the FAR by identifying the value that maximised Youden’s J statistic (sum of sensitivity and specificity) on time-dependent receiver operating characteristic (ROC) curve analysis for MACCEs to ensure an optimum balance between sensitivity and specificity in our models. Cox proportional hazard models were used to estimate the association between FAR and MACCEs. Survival probabilities are estimated by the *Kaplan*–*Meier* *method*. By adding the FAR and CACS to a baseline model consisting of traditional risk factors, we assessed the improvement in model performance and discrimination and risk classification by (1) comparing the C statistic (area under the curve [AUC]) of the two nested models (from time-dependent ROC analysis), (2) doing a likelihood ratio test (in Cox regression models), and (3) calculating the integrated discrimination improvement (IDI) and net reclassification improvement (NRI) indices for censored data. We used bootstrapping with 200 replications to calculate 95% CIs for AUC, NRI, and IDI [19].

We did statistical analyses with the SPSS program (version 22.0; SPSS) and R version 3.4.0 (survival, PredictABEL, and nricens). All tests were two-sided and α was set at 0.05.

## Results

### Baseline characteristics

A total of 4946 patients were enrolled in the final analysis. The median follow-up time was 42.20 months.Baseline characteristics are presented in Table 1. MACCEs were reported in 234 of the patients. Overall, patients with MACCEs were older than patients with no MACCEs during the follow-up period. In addition, they had higher levels of creatinine, fasting blood glucose, and fibrinogen, while lower levels of GER, TG, HDL-C, and albumin. Furthermore, CACS and FAR were significantly higher than those in the non-MACCEs group (both *p* = 0.001). A total of 2809 patients (56.8%) were categorized as FAR-H (> = 0.0817), and 2137 patients (43.2%) were categorized as FAR-L group (< 0.0817) according to the optimal cutoff value. In addition,comparison of baseline characteristics stratified by low or high FAR, participants and non-participants in supplemental Table 1 and 2.

**Table 1:** Comparison of baseline characteristics stratified by the primary endpoint

Variable	ALL (4946)	Non-MACCEs (4712)	MACCEs (234)	*P* value
Age,years	62 (55–71)	62 (54–70)	65 (58–74)	0.001
Male, n (%)	3182 (64.3)	3021(64.1)	161(68.8)	0.144
Hypertension,n (%)	3415 (69.0)	3249 (69.0)	166 (70.9)	0.521
DM,n (%)	1532 (31.0)	1451 (30.8)	81 (34.6)	0.217
Family history of CAD, n (%)	693(14.0)	663(14.1)	30(12.8)	0.591
Smoking,n (%)	1223 (24.7)	1175 (24.9)	48 (20.5)	0.126
SBP,mmHg	129 (120–140)	129 (120–140)	129 (120–140)	0.713
DBP,mmHg	78 (70–84)	78 (70–84)	78 (70–83)	0.756
BMI, kg/m2	25.71 (23.49–28.4)	25.71 (23.44–28.41)	25.61 (23.71–28.09)	0.739
Creatinine,µmol/L	69.0 (58.97–80.00)	59 (58.6–79.68)	71 (62–84.76)	0.004
UA, mg/dL	320 (266–376.21)	320 (266–376)	325 (267–382.24)	0.268
eGFR, mL/min/1.73 m^2^	91.28 (69.45–117.50)	91.84 (70.02–118.08)	83.702 (62.8–109.23)	0.001
FBG, mmol/L	5.37 (4.75–6.82)	5.35 (4.75–6.78)	5.54 (4.82–7.64)	0.024
TG, mmol/L	1.62 (1.13–2.43)	1.62 (1.13–2.44)	1.47 (1.07–2.23)	0.037
TC, mmol/L	4.18 (3.52–4.88)	4.18 (3.52–4.87)	4.20(3.35–4.98)	0.657
HDL-C, mmol/L	1.11 (0.93–1.32)	1.11 (0.93–1.33)	1.05 (0.87–1.26)	0.002
LDL-C, mmol/L	2.72 (2.12–3.33)	2.72(2.13–3.32)	2.74 (2.07–3.50)	0.466
Total protein, g/L	67.7 (64.3–72.00)	67.7 (64.3–72.00)	67.45 (63.8–71.9)	0.71
Albumin,g/L	41.2(38.64–43.70)	41.2(38.7–43.73)	40.55(37.50–43.10)	0.001
Fibrinogen, g/L	3.27(2.82–3.69)	3.26 (2.81–3.68)	3.37 (3.03–3.83)	0.001
FAR	0.0787(0.0671–0.0913)	0.0785 (0.0669–0.0909)	0.0833 (0.0727–0.0974)	0.001
CACS	75.55(18.20–259.73)	73.20 (17.5–253.3)	156.40 (39.2–498.1)	0.001
Medication				
Aspirin, n (%)	2365(47.8)	2260(48.0)	105(44.9)	0.356
β-blocker, n (%)	1528(30.9)	1465(31.1)	63 (26.9)	0.178
ACEI/ARB, n (%)	1357(27.4)	1317(27.9)	40(17.1)	0.001
Statins, n (%)	2639(53.4)	2525(53.6)	114 (48.7)	0.145
CCB, n (%)	1305 (26.4)	1268 (26.9)	37(15.8)	0.001

Values are median (25th to 75th percentile) or number (%)

*MACCEs* major adverse cardiovascular and cerebral events, *FAR* fibrinogen-to-albumin ratio, *BMI* body mass index, *DM* diabetes mellitus, *CAD* coronary artery disease, *FBG* fasting blood glucose, *TG* triglyceride, *TC* total cholesterol, *HDL-C* high-density lipoprotein cholesterol, *LDL-C* low-density lipoprotein cholesterol, *eGFR* estimated glomerular filtration rate, *CACS* Coronary calcification score, *ACEI* angiotensin converting enzyme inhibitors, *ARB* angiotensin receptor blockers, *CCB* calcium-channel blocker

*p*-values < 0.05 were considered significant

**Table 2:** Univariate and multivariate COX regression analysis for predicting MACCEs

Variables	Univariate HR(95% CI)	*P*	Multivariate HR (95% CI)	*P* value
Age, years	1.026 (1.013–1.038)	0.001	1.015 (1.001–1.028)	0.03
LDL-C	1.091 (0.950–1.253)	0.219		
SBP	1.002 (0.995–1.009)	0.537		
DBP	1.001 (0.990–1.013)	0.844		
Creatinine,µmol/L	1.001(0.999–1.004)	0.288		
eGFR, mL/min/1.73 m^2^	0.996(0.993–1.000)	0.059		
FBG, mmol/L	1.055(1.017–1.095)	0.005	1.043(1.006–1.083)	0.024
TG, mmol/L	0.986(0.940–1.034)	0.561		
FAR	1.475(1.139–1.910)	0.003	1.355(1.042–1.763)	0.023
CACS	1.572(1.347–1.835)	0.001	1.470(1.250–1.7727)	< 0.001
Aspirin	0.884 (0.683–1.144)	0.350		
β-blocker	0.856 (0.641–1.144)	0.293		
ACEI/ARB	0.564 (0.401–0.793)	0.001	0.575 (0.409–0.808)	0.001
Statins	0.846 (0.654–1.093)	0.846		
CCB	0.469 (0.330–0.666)	< 0.001	0.469 (0.330–0.667)	< 0.001

Adjust variables were Age, FBG, FAR, CACS, ACEI/ARB, and CCB

*FAR* fibrinogen-to-albumin ratio, *FBG* fasting blood glucose, *TG* triglyceride, *eGFR* estimated glomerular filtration rate, *CACS* Coronary calcification score, *ACEI* angiotensin converting enzyme inhibitors, *ARB* angiotensin receptor blockers, *CCB* calcium-channel blocker

### Relationship between FAR and CACS

Linear regression analysis was conducted to evaluate the correlation between FAR and CACS (Supplemental Table 3 and Fig. 1). The results showed that CACS (R2 = 0.004, Standard β = 0.066, *P* < 0.001) was positively associated with FAR in patients with chest pain. Furthermore, both in the MACCEs and in the non-MACCEs, there was also a positive relationship between FAR and CACS (R2 = 0.024, Standard β = 0.167 *P* = 0.011; R2 = 0.003, Standard β = 0.053, *P* < 0.001, respectively).

**Table 3:** Baseline FAR and prediction of MACCEs

Variables	FAR	Events(n/total)	Event rate(%)	Crude HR(95% CI)	*P* value	Adjusted HR(95% CI)	*P* value
MACCES	Low	104/2809	3.7	Reference	—		
	High	130/2137	6.1	1.475(1.139–1.910)	0.003	1.371 (1.053–1.786)	0.019
Revascularization	Low	49/2809	1.7	Reference	—		
	High	49/2137	2.3	1.186(0.798–1.764)	0.398	1.086 (0.792–1.764)	0.298
Non-fatal MI	Low	6/2809	0.2	Reference	—		
	High	9/2137	0.4	1.743(0.619–4.905)	0.292	1.343 (0.629–2.905)	0.392
All-cause death	Low	48/2809	1.7	Reference	—		
	High	73/2137	3.4	1.78(1.236–2.564)	0.002	1.411 (0.973–2.045)	0.069
Cardiac Death	Low	29/2809	1.0	Reference	—		
	High	57/2137	2.7	2.323(1.485–3.634)	< 0.001	1.863 (1.182–2.937)	0.007
Stroke	Low	20/2809	0.7	Reference	—		
	High	15/2137	0.7	0.873(0.446–1.707)	0.691	0.773 (0.446–1.207)	0.591

Adjust: age, smoking,male, FBG,BMI, DM, hypertension, CACS

*MACCEs* major adverse cardiovascular and cerebral events, *FAR* fibrinogen-to-albumin ratio, *MI* myocardial infarction, *FBG* fasting blood glucose, *BMI* body mass index, *DM* diabetes mellitus, *CACS* Coronary calcification score

### The poor prognosis associated with FAR-H

Univariate Cox regression analysis showed that age (crude HR 1.026, 95% CI 1.013–1.038;*P* = 0.001), FBG(crude HR 1.055, 95% CI 1.017–1.095;*P* = 0.005), FAR (crude HR 1.457, 95% CI 1.139–1.910;*p* = 0.003),ACEI/ARB(crude HR 0.564, 95% CI 0.401–0.793;*P* = 0.001),CCB(crude HR 0.469, 95% CI 0.330–0.666;*P* < 0.001) and CACS (crude HR 1.572, 95% CI 1.347–1.835;*p* = 0.001)were associated with MACCEs. Furthermore, multivariate Cox regression showed that age (adjusted HR 1.015 95% CI 1.001–1.028;*p* = 0.03), FAR (adjusted HR 1.355 95% CI 1.042–1.763;*p* = 0.023),FBG (adjusted HR 1.043 95% CI 1.006–1.083;*p* = 0.024)and CACS(adjusted HR 1.470 95% CI 1.250–1.727;*p* < 0.001) were the independent risk factors for MACCEs (Table 2).

**Fig. 2:**
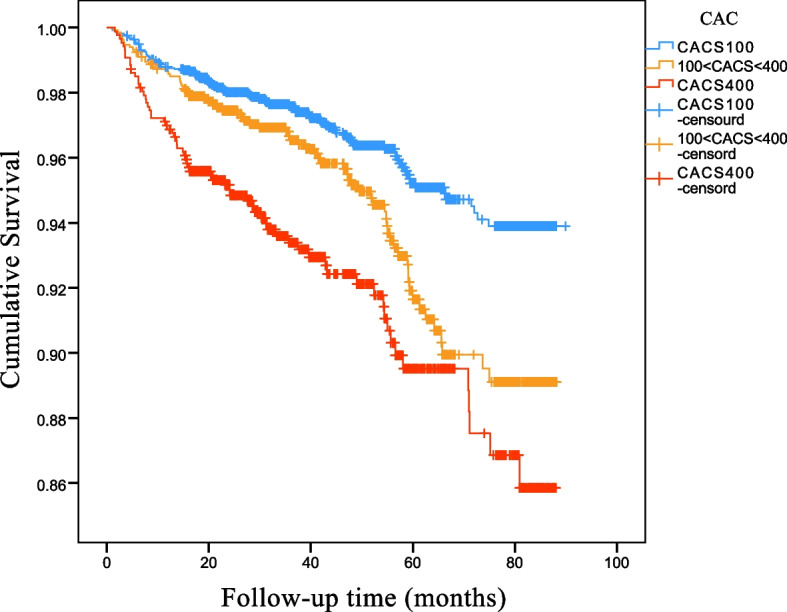
Kaplan–Meier curves showing from MACCES according to the Coronary calcification score

**Fig. 3:**
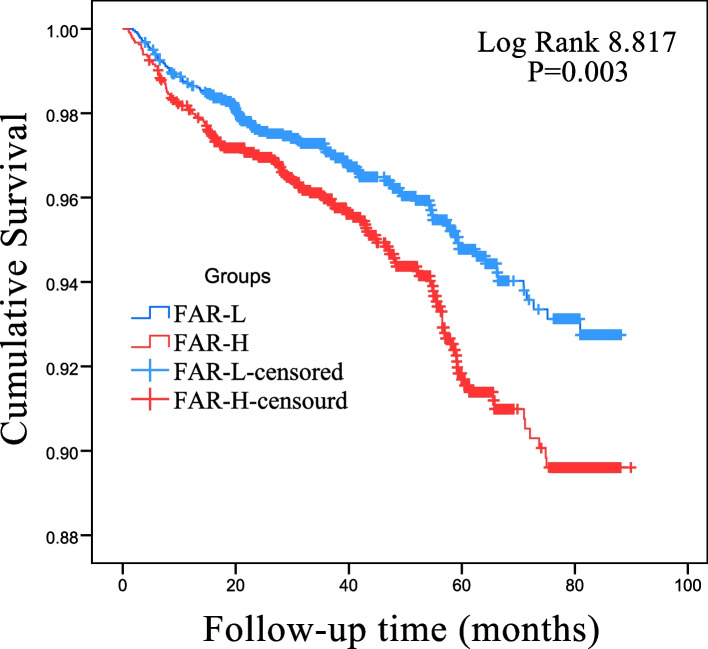
Kaplan Meier curves showing form MACCES according to the Fibrinogen-to-Albumin Ration

Univariable analysis showed that FAR-H was associated with higher risks of MACCEs, all-cause death, and cardiac death(crude HR 1.475, 95% CI 1.139–1.910,*p* = 0.0003; crude HR 1.78, 95% CI 1.236–2.564,*p* = 0.002; crude HR 2.323, 95% CI 1.485–3.634,*p* < 0.001) (Table 3). After adjusting for age, smoking, male, Fasting glucose, BMI, diabetes mellitus, hypertension, CACS, the FAR-H group had a higher risk of cardiac death (adjusted HR 1.863(1.182–2.937) *P* = 0.007) and MACCEs (adjusted HR 1.371(1.053–1.786) *P* = 0.019). In Fig. 2, there are significant differences in the prognosis of patients in different CACS groups. The higher the CACS, the worse the prognosis.The Kaplan–Meier curve showed that the prognosis of the FAR-H group was significantly poorer than that of the FAR-L group (Fig. 3).

### Relationship between FAR combined with CACS and primary endpoints

We calculated the number of events and cumulative event incidence in patients by high and low FAR values and CACS of ≤ 100, 100 < CACS ≤ 400, and > 400. Cox regression showed that the rate for MACCEs increased with the FAR level and further increased with the severity of coronary artery calcification, especially in patients of CACS > 400, yielding a higher event risk in those with the FAR-H (HR 1.972, 95% CI 1.199–3.245, *P* = 0.007) (Table 4). The similar trends were seen for all-cause and cardiac deaths. In the CACS 100 < CACS ≤ 400 group, the incidence of MACCEs (HR: 1.664, 95% CI: 1.199–3.245, *P* = 0.007), all-cause death (HR 1.917, 95% CI 1.009–3.639, *P* = 0.047) and cardiac death (HR 2.693, 95% CI 1.232–5.887, *P* = 0.0013) was higher and statistically significant in patients with FAR-H.

**Table 4:**
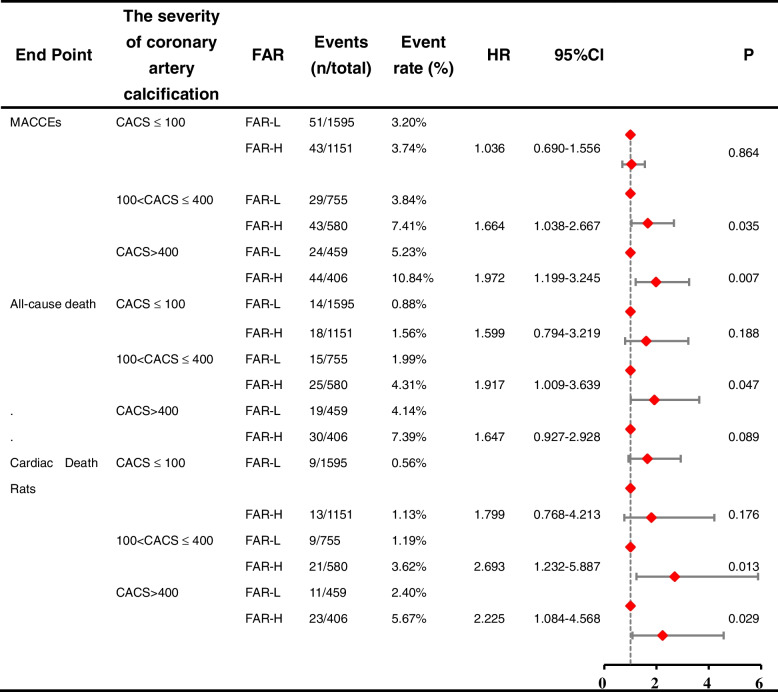
Evaluation of Predictive Models for MACCEs

*FAR* fibrinogen-to-albumin ratio, *CACS* Coronary calcification score

### Predictive value of FAR combined with CACS for MACCEs

**Fig. 4:**
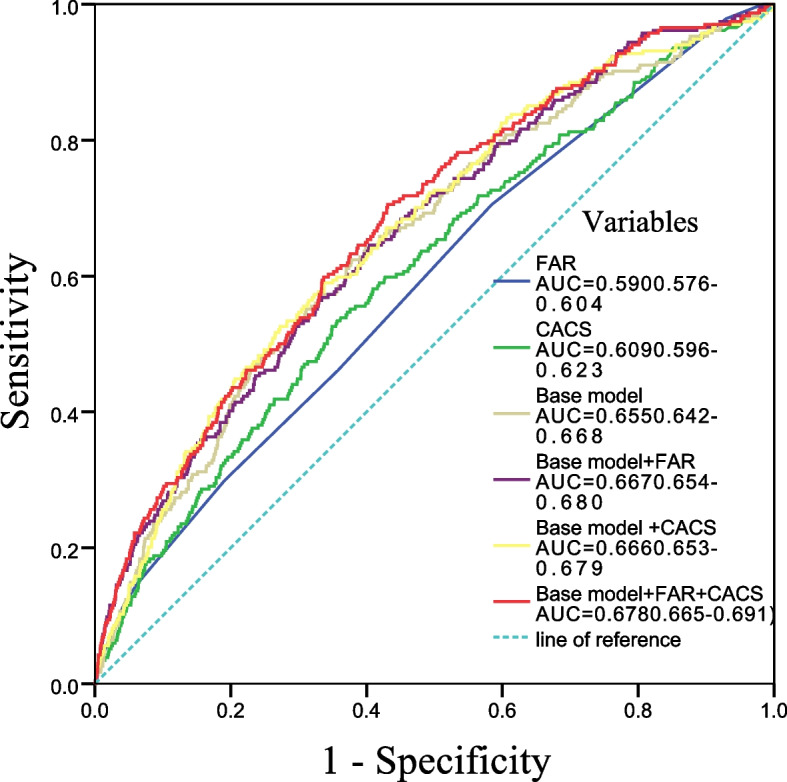
Receiver operating characteristic curve showing from MACCES according to the different Variables

Additionally, we tested the performance of FAR and CACS for predicting MACCEs (Table 5 and Fig. 4). The AUC of FAR and CACS were 0.590 (95%CI 0.576–0.604) and 0.609 (95%CI 0.596–0.623), respectively. The AUC for the base model was 0.655 (95% CI 0.642–0.668) for MACCEs. After adding FAR and CACS to the base model the AUC was 0.667 (95% CI, 0.654–0.680), and 0.666 (95% CI, 0.653–0.679) for MACCEs respectively. The combining FAR and CACS with the base model the AUC was raised to 0.678(95% CI, 0.665–0.691). Combining FAR and CACS significantly improved MECCEs risk stratification, contributing to net reclassification improvement ( NRI 0. 122, 95% CI 0.054–0.198, *P* < 0.001) and integrated discrimination improvement(IDI 0.011, 95% CI 0.006–0.017, *P* < 0.001).

**Table 5:** FAR predicting MACCEs among different severity of coronary artery calcification

Variables	AUC-Statistic	*P* value	NRI	*P* value	IDI	*P* value
FAR	0.590 (0.576–0.604)	< 0.001				
CACS	0.609 (0.596–0.623)	< 0.001				
Base model	0.655 (0.642–0.668)	< 0.001	Reference	—	Reference	—
Base model + FAR	0.667 (0.654–0.680)	< 0.001	0.087(0.026–0.139)	0.002	0.007(0.003–0.011)	< 0.001
Base model + CACS	0.666 (0.653–0.679)	< 0.001	0.063(0.012–0.111)	0.017	0.005(0.001–0.009)	< 0.001
Base model + FAR + CACS	0.678 (0.665–0.691)	< 0.001	0. 122(0.054–0.198)	< 0.001	0.011(0.006–0.017)	< 0.001

Base model: age, sex, BMI, smoking,FBG, Hypertension, DM,Aspirin, ACEI/ARB, β-blocker, Statins, and CCB

*MACCEs* major adverse cardiovascular and cerebral events, *FAR* fibrinogen-to-albumin ratio, *FBG* fasting blood glucose, *BMI* body mass index, *DM* diabetes mellitus, *CACS* Coronary calcification score, *ACEI* angiotensin converting enzyme inhibitors, *ARB* angiotensin receptor blockers, *CCB* calcium-channel blocker

## Discussion

In this real, retrospective, observational study, we investigated the association between different fibrinogen-to-albumin ratio levels and CACS with adverse outcomes in patients with chest pain in a large cohort from China. Our data showed that suspected CAD patients with a higher level of FAR have a significantly increased risk of long-term MACCEs,compared with patients with a lower level of FAR. The results showed that CACS was positively associated with FAR in patients with suspected CAD. Furthermore, suspected CAD patients with FAR-H and higher CAC burden also have a higher risk of MACCEs and cardiac death than patients with FAR-L and burden of CAC. Multivariate COX regression analysis also showed that FAR was an independent risk factor for MACCEs. In addition, with the addition of FAR and CACS, the base model’s performance of prediction has also been improved.

We found a higher incidence of MACCEs in patients with 100 < CACS ≤ 400 and CACS > 400 combined with high FAR, suggesting that FAR could identify patients at higher risk of MACCEs in patients with moderate to severe coronary calcification and optimizing the risk assessment. For the CAD patients with high FAR at risk of MACCE, we should take more aggressive treatment and more attention. In addition, Risk factors such as blood pressure, blood lipids and blood glucose should be more strictly managed.

Coronary CTA could assess the degree of vessel stenosis, among which severe stenosis (70–99% luminal stenosis) requires invasive coronary angiography to evaluate whether to perform coronary revascularization. When there was evidence of nonobstructive (10 to 50% cross-sectional luminal stenosis) or CAD the CTA, the physician should be prompted to prescribe preventive therapies [20]. In addition, noninvasive and intracoronary imaging has been used to analyze the association between plaque components and serum biomarkers [21, 22]. Future study may need to explore the relationship between FAR and plaque features using non-invasive and intra-coronary imaging modalities.

Atherosclerosis is considered a chronic inflammation in which inflammatory cells infiltrate lesions and participate in plaque progression and thrombosis, leading to acute coronary syndrome [23]. Fibrinogen plays a key role in the inflammatory and clotting cascade [11, 24]. The relationship between fibrinogen and inflammation has been reported in several kinds of literature, in which high fibrinogen level was associated with adverse outcomes of CAD [25, 26]. One of these studies demonstrated that plasma fibrinogen levels were an independent predictor of the severity and severity of CAD, estimated based on the number of vessels affected and the Gensini score [25]. Peng et al. showed that fibrinogen ≥ 3:17 g/L was an independent predictor of all-cause mortality and cardiac mortality in patients with CAD at admission [26]. Albumin, a major component in maintaining the osmotic pressure of plasma colloids, involves the acute inflammatory response [8, 9]. Many observational studies and meta-analyses have shown that serum albumin levels were inversely associated with cardiovascular outcomes [27–29]. Low serum albumin levels at admission have been reported to predict the absence of reflux after direct percutaneous coronary intervention in STEMI patients [27]. Rezkalla et al. showed that hypoproteinemia may aggravate ischemia–reperfusion injury in patients without coronary artery reflow [28]. Both plasma fibrinogen and albumin are useful inflammatory biomarkers and are strongly associated with cardiovascular events. There has been little research on their value in patients with chest pain. More research is needed to further assess their correlation and whether they can help identify individuals at high risk for patients with chest pain.

FAR is a promising serum measure that predicts MACE more accurately and specifically than fibrinogen and albumin by themselves. According to Sapmaz [30] and colleagues, significantly higher FAR values may be linked to a higher risk of cardiovascular events. They also proposed that hemorheological substances like albumin and fibrinogen, which can affect blood viscosity, are crucial in the development of pathological vascular thrombosis. The levels of albumin and fibrinogen may need to be measured in tandem in order to fully understand the pathophysiology of cardiovascular events. Several articles have been published on the prognostic value of FAR in different clinical settings [31, 32]. Karahan et al. [31] found that FAR was significantly correlated with SYNTAX score in predicting CAD severity in STEMI patients. Xiao et al. [32] analyzed 475 patients with ST-segment elevation myocardial infarction (STEMI) who received the percutaneous coronary intervention and found that FAR was an independent predictor of percutaneous coronary intervention in STEMI patients. More importantly, a large body of evidence suggested that elevated CAC levels are closely associated with an increased risk of MACE in stable, symptomatic patients with suspected CAD, with a higher CAC score associated with an increased risk [6, 33]. One meta-analysis included 19 studies evaluating the prognostic value of CAC in predicting the risk of MACE in patients with suspected stable CAD [6]. The final results showed that increased coronary artery calcium levels are strongly associated with an increased risk of major adverse cardiac events and that Coronary CTA should aid clinical decision-making in a significant number of stable patients with chest pain. Our study showed that FAR was positively associated with CACS, and the combination of both could improve the predictive power for adverse cardiovascular outcome events in patients with chest pain. It is of great significance to study the effect of the combined application of FAR and CAC on long-term MACCEs in patients with chest pain. However, there is little literature on the relationship between FAR, different CACS, and cardiovascular events.

Our study is the first to demonstrate that a higher level of FAR combined with CACS is strongly associated with an increased risk of long-term MACCEs. In addition, patients with a higher level of FAR and a heavier CAC burden had a higher risk of all-cause mortality and cardiac death than patients with a lower level of FAR and a lighter burden of CAC. In the traditional risk factor model, adding FAR and CACS can significantly improve the performance of prediction of MACCEs. Interestingly, we observed no significant differences between groups in the risk of other components of MACCEs, such as Non-fatal MI, stroke, or revascularization, as observed in other studies [13]. This is worth further exploring in future studies.

Another issue to be discussed is the potential mechanism by which FAR and CAC are associated with poor prognosis. First, previous studies have confirmed that fibrinogen upregulates IL-1, TNF-α, and other proinflammatory cytokines expression, thereby inducing vascular inflammation and endothelial dysfunction [34, 35]. This is followed by monocyte or macrophage adhesion, which stimulates vascular smooth muscle cell proliferation and migration, ultimately leading to atherosclerotic plaque formation and vulnerability. Furthermore, previous literature suggests that higher concentrations of plasma fibrinogen may increase blood viscosity and peripheral resistance [36], thereby increasing the risk of thrombotic and ischemic events during follow-up. Second, some basic studies have proved that serum albumin at physiological concentrations can inhibit the expression of vascular cell adhesion molecule-1, increase the scavenging of oxygen free radicals, and ultimately attenuate inflammatory responses, suggesting that albumin has protective anti-inflammatory effects [8]. Third, inflammation is a common precursor to CAC and atherosclerosis [37, 38]. Fourth, CACS is associated with the severity of coronary stenosis and is closely related to the prognosis of patients with CAD [5, 6]. In addition, further research is needed to elucidate the possible mechanisms.

The current understanding of natural history of coronary atherosclerosis includes subclinical plaque healing as a cause of lesion progression [39]. Inflammatory cells infiltrate plaques and participate in plaque progression and thrombosis, leading to acute coronary syndrome [23, 40]. When plaque rupture or erosion occurs in a prothrombotic milieu, subocclusive or occlusive thrombosis results, causing a symptomatic acute coronary event; otherwise, if thrombosis resisting factors prevail, thrombus formation is contained, and plaque healing occurs [23, 41]. Plaque erosion is due to endothelial damage or denudation and thrombosis in the absence of fibrous cap formation [40]. Fibrin is involved in thrombosis and can reflect the level of inflammation [11]. Albumin is associated with inhibition of platelet activation and inflammation [8]. A high FAR may represent a greatly increased risk of thrombosis during plaque rupture or erosion [42]. To sum up, FAR may reflect the environmental state before thrombosis to a certain extent.

### Study limitations

The study has several limitations. Firstly, FAR levels were calculated only at baseline. The dynamics of this new biomarker were absent during follow-up. The dynamics of this new biomarker were absent during follow-up. Secondly, due to the nature of observational studies, potential confounders cannot be fully adjusted. Further randomized clinical trials are needed to confirm our findings. Thirdly, there is a lack of data on detailed medication outcomes, including the intensity and compliance rate of lipid-lowering drugs and the use of hypoglycemic agents. This study was a single-center study. All patients had chest pain confirmed by CTAs, so the applicability of the conclusions of this study needs further study to examine patients with no clinical symptoms. Finally, the study involved Chinese patients, so these results need to be replicated in other ethnic groups.

## Conclusion

In this real-world study, a higher level of FAR and heavier CAC burden was associated with worse outcomes among patients with suspected CAD. Our results may help guide treatment allocation in clinical practice, personalize the use of preventive therapy by conducting more accurate risk assessments and inform the updating of future guidelines.

## Supplementary Information

**Additional file 1:** **Supplemental Table 1. **Comparison of baseline characteristics stratifiedby low or high FAR. **Supplemental Table 2.** Comparison of baselinecharacteristics of participants and non-participants due to exclusioncriteria. **Supplemental Table 3.** Correlation analysis between CACS and FAR inpatients with MACCE, Non-MACCE and whole. **Supplemental Figure 1. **linear regressionwith scatter dots about FAR and CACS. 

## Data Availability

The datasets used and/or analysed during the current study are available from the corresponding author on reasonable request.
