# Supplementary material for: Prognostic value of fibrinogen-to-albumin ratio combined with coronary calcification score in patients with suspected coronary artery disease
Source: BMC Cardiovasc Disord. 2023 Apr 4;23:181. doi: 10.1186/s12872-023-03193-z (PMC10071697; doi:10.1186/s12872-023-03193-z)
Supplement: Supplementary file 1 — Additional file 1: Supplemental Table 1. Comparison of baseline characteristics stratifiedby low or high FAR. Supplemental Table 2. Comparison of baselinecharacteristics of participants and non-participants due to exclusioncriteria. Supplemental Table 3. Correlation analysis between CACS and FAR inpatients with MACCE, Non-MACCE and whole. Supplemental Figure 1. linear regressionwith scatter dots about FAR and CACS. [file 12872_2023_3193_MOESM1_ESM.docx]

**Supplemental Table 1** Comparison of baseline characteristics stratified by low or high FAR

| **Variable** | **ALL （4946)** | **FAR-L (2807)** | **FAR-H （2137）** | **P value** |
| --- | --- | --- | --- | --- |
| Age ,years | 62（55-71） | 60（53-69） | 65（57-72） | <0.001 |
| Male , n (%) | 3182（64.3) | 1954(69.6) | 1228(57.5) | <0.001 |
| Hypertension ,n (%) | 3415（69.0） | 1917（68.2） | 1498（70.1） | 0.163 |
| DM ,n (%) | 1532（31.0） | 860（30.6） | 672（31.7) | 0.532 |
| Family history of CAD , n (%) | 693(14.0) | 393(14.0) | 300(14.0) | 0.962 |
| Smoking ,n (%) | 1223（24.7） | 760（27.1） | 463（21.7） | <0.001 |
| SBP ,mmHg | 129（120-140） | 129（120-140） | 129（120-140） | 0.462 |
| DBP ,mmHg | 78（70-84） | 78（70-84） | 78（70-83） | <0.001 |
| BMI, kg/m2 | 25.71（23.49-28.4） | 25.76（23.53-28.37） | 25.64（23.21-28.42） | 0.499 |
| Creatinine ,µmol/L | 69（58.97-80） | 97.14（74.40-122.83） | 84.97（64.41-110.75） | 0.136 |
| UA , mg/dL | 320（266-376.21） | 325（274.00-383.05） | 312.3（256.33-366.00） | <0.001 |
| eGFR, mL/min/1.73 m^2^ | 91.28（69.45-117.50） | 91.84（70.02-118.08） | 83.702（62.8-109.23） | <0.001 |
| FBG, mmol/L | 5.37（4.75-6.82） | 5.31（4.74-6.57） | 5.43（4.77-7.16） | <0.001 |
| TG , mmol/L | 1.62（1.13-2.43） | 1.69（1.16-2.72） | 1.53（1.09-2.18） | <0.001 |
| TC, mmol/L | 4.18（3.52-4.88） | 4.18（3.52-4.91） | 4.18(3.51-4.83) | 0.242 |
| HDL-C, mmol/L | 1.11（0.93-1.32） | 1.11（0.93-1.34） | 1.11（0.91-1.30） | 0.002 |
| LDL-C, mmol/L | 2.72（2.12-3.33） | 2.72(2.12-3.33） | 2.74（2.13-3.33） | 0.471 |
| Total protein , g/L | 67.7（64.3-72.00） | 68.2（64.80-72.5） | 67.17（63.50-71.3) | <0.001 |
| Albumin ,g/L | 41.2(38.64-43.70) | 42.60(40.40-45.04) | 39.40(36.80-41.50) | <0.001 |
| Fibrinogen , g/L | 3.27(2.82-3.69） | 2.90（2.58-3.19） | 3.74（3.48-4.00） | <0.001 |
| FAR | 0.079(0.067-0.091) | 0.069（0.061-0.076) | 0.093(0.087-0.104) | <0.001 |
| CACS | 75.55(18.20-259.73) | 70.40（15.85-244.40） | 82.10（21.25-288.45） | <0.001 |
| Medication |  |  |  |  |
| Aspirin, n (%) | 2365(47.8) | 1352(48.1) | 1013(47.4) | 0.612 |
| β-blocker, n (%) | 1528(30.9) | 853 (30.4) | 675（31.6） | 0.358 |
| ACEI/ARB, n (%) | 1357(27.4) | 757(26.9) | 600 (28.1) | 0.379 |
| Statins, n (%) | 2639(53.4) | 1533(54.6) | 1106(51.8） | 0.049 |
| CCB, n (%) | 1305（26.4） | 720（25.6） | 585(27.4) | 0.168 |

Values are median (25th to 75th percentile) or number (%).MACCEs major adverse cardiovascular and cerebral events, FAR fibrinogen-to-albumin ratio, BMI body mass index, DM diabetes mellitus, CAD coronary artery disease, FBG fasting blood glucose, TG triglyceride, TC total cholesterol, HDL-C high-density lipoprotein cholesterol, LDL-C low-density lipoprotein cholesterol, eGFR estimated glomerular filtration rate, CACS Coronary calcification score, ACEI angiotensin converting enzyme inhibitors, ARB angiotensin receptor blockers, CCB calcium-channel blocker

**Supplemental Table 2** Comparison of baseline characteristics of participants and non-participants due to exclusion criteria.

| **Variable** | **Non-participants (7947)** | **Participants  （4946）** | **P value** |
| --- | --- | --- | --- |
| Age ,years | 63（55-72） | 62（55-71） | <0.001 |
| Male , n (%) | 5499(69.2) | 3182（64.3) | <0.001 |
| Hypertension ,n (%) | 5511（69.3） | 3415（69.0） | 0.719 |
| DM ,n (%) | 2609（32.8） | 1532（31.0） | 0.028 |
| Family history of CAD , n (%) | 1041(13.1) | 693(14.0) | 0.140 |
| Smoking ,n (%) | 2663（33.5） | 1223（24.7） | <0.001 |
| SBP ,mmHg | 128（119-140） | 129（120-140） | 0.183 |
| DBP ,mmHg | 76（70-82） | 78（70-84） | 0.025 |
| BMI, kg/m2 | 26.23（23. 74-28.73） | 25.71（23.49-28.4） | 0.008 |
| Creatinine ,µmol/L | 97.14（74.40-122.83） | 69（58.97-80） | <0.001 |
| UA , mg/dL | 325（270.25-389.93） | 320（266-376.21） | 0.097 |
| eGFR, mL/min/1.73 m^2^ | 92.95（81.9-101.928） | 91.28（69.45-117.50） | 0.005 |
| FBG, mmol/L | 5.33（4.70-6.73） | 5.37（4.75-6.82） | 0.073 |
| TG , mmol/L | 1.60（1.13-2.54） | 1.62（1.13-2.43） | <0.001 |
| TC, mmol/L | 3.76（3.07-4.53） | 4.18（3.52-4.88） | <0.001 |
| HDL-C, mmol/L | 1.08（0.90-1.29） | 1.11（0.93-1.32） | <0.001 |
| LDL-C, mmol/L | 2.42(1.82-3.12） | 2.72（2.12-3.33） | <0.001 |
| Total protein , g/L | 68.3（64.40-73.00） | 67.7（64.3-72.00） | 0.796 |
| Albumin ,g/L | 41.90(38.90-41.90) | 41.2(38.64-43.70) | 0.863 |
| Fibrinogen , g/L | 3.09（2.61-3.56） | 3.27(2.82-3.69） | <0.001 |
| FAR | 0.067（0.053-0.079) | 0.079(0.067-0.091) | 0.217 |
| CACS | 70.40（15.85-244.40） | 75.55(18.20-259.73) | <0.001 |

Abbreviations as in Table 1 and Table S1.

**Supplemental Table 3** Correlation analysis between CACS and FAR in patients with MACCE, Non-MACCE and whole

| **Variables** | **Adjusted R^2^** | **Coefficient** | **Standard β** | **P value** |
| --- | --- | --- | --- | --- |
| Whole |  |  |  |  |
| CACS | 0.004 | 0.002 | 0.066 | <0.001 |
| Non-MACCE |  |  |  |  |
| CACS | 0.003 | 0.002 | 0.053 | <0.001 |
| MACCE |  |  |  |  |
| CACS | 0.024 | 0.006 | 0.167 | 0.011 |

MACCEs major adverse cardiovascular and cerebral events ,FAR fibrinogen‑to‑albumin ratio , CACS Coronary calcification score

**Supplemental Figure 1** linear regression with scatter dots about FAR and CACS

FAR fibrinogen‑to‑albumin ratio , CACS Coronary calcification score
